# The Influence of Booster Shot and SARS-CoV-2 Infection on the Anti-Spike Antibody Concentration One Year after the First COVID-19 Vaccine Dose Administration

**DOI:** 10.3390/vaccines11020278

**Published:** 2023-01-28

**Authors:** Jakub Swadźba, Tomasz Anyszek, Andrzej Panek, Agnieszka Chojęta, Anna Piotrowska-Mietelska, Emilia Martin

**Affiliations:** 1 Medical Faculty, Andrzej Frycz Modrzewski Krakow University, 30-705 Krakow, Poland; 2 Medical Department, Diagnostyka S.A., 31-864 Krakow, Poland

**Keywords:** SARS-CoV-2 antibodies, COVID-19 vaccination, booster dose, humoral immunity, Comirnaty, CLIA, immunoassay

## Abstract

This study pictures the humoral response of 100 vaccinees to Pfizer/BioNTech COVID-19 vaccine over a year, with particular focus on the influence of a booster shot administered around 10 months after the primary immunization. The response to the vaccination was assessed with Diasorin’s SARS-CoV-2 TrimericSpike IgG. Abbott’s SARS-CoV-2 Nucleocapsid IgG immunoassay was used to identify SARS-CoV-2 contact, even asymptomatic. In contrast to the gradual decline of the anti-spike IgG between 30 and 240 days after the first dose, an increase was noted between days 240 and 360 in the whole cohort. However, a statistically significant rise was seen only in boosted individuals, and this effect of the booster decreased over time. An increase was also observed in non-boosted but recently infected participants and a decrease was reported in non-boosted, non-infected subjects. These changes were not statistically significant. On day 360, a percentage of new SARS-CoV-2 infections was statistically lower in the boosted vs. non-boosted subgroups. The booster immunization is the most efficient way of stimulating production of anti-spike, potentially neutralizing antibodies. The response is additionally enhanced by the natural contact with the virus. Individuals with a low level of anti-spike antibodies may benefit the most from the booster dose administration.

## 1. Introduction

On 21 December 2020, the European Commission granted a conditional marketing authorization for the first coronavirus disease 2019 (COVID-19) vaccine. In less than a week, the first vaccination was performed in Poland. Inoculation was at first offered to the healthcare workers, then the elderly, followed by the entire adult population in May 2021. Similar vaccination strategies were implemented all over Europe and resulted in a milestone of 70% of the adult EU population being fully vaccinated on 31 August 2021 [1].

The coveted vaccine-induced COVID-19 protection includes several immune mechanisms. Severe acute respiratory syndrome coronavirus 2 (SARS-CoV-2) specific antibody production is the most feasible to detect and assess. Although no recommendations on routine testing of the vaccine immunogenicity in the general population of vaccinees have been released [2,3], with the advent of the widespread COVID-19 vaccinations, a demand for vaccination-induced anti-SARS-CoV-2 antibodies testing sprouted [4,5]. Since most commonly used COVID-19 vaccines are based on SARS-CoV-2 spike (S) protein, antibodies of such specificity are used as a marker of vaccine immunogenicity. They are also induced during the natural contact with the virus and, therefore, may indicate SARS-CoV-2 protection acquired through both infection and vaccination. Spike-specific immunoglobulins block the virus interaction with ACE2 receptor on human cells and have been shown to confer neutralizing activity against SARS-CoV-2 [6,7]. Unfortunately, their titers have been shown to decrease substantially over a few months after the vaccination [8,9,10]. Similar phenomenon of antibody waning has been observed over a natural SARS-CoV-2 infection course [11,12].

The protective function of anti-nucleocapsid SARS-CoV-2 antibodies is less pronounced, so rather than as a marker of immunity they are used in epidemiological studies. Their presence indicates a recent infection [13], as the titers wane even quicker than anti-spike immunoglobulins. Nucleocapsid sequence is not included in mRNA vaccines, so antibodies of this specificity may be utilized as markers of SARS-CoV-2 natural infection in both the vaccinated and non-vaccinated population [14].

In October 2021, the European Medicines Agency recommended booster dose administration 6 months after the full vaccination course in the adult population [15], outlining that the extra dose led to a rise in antibody levels in adults whose antibody levels were waning.

This recommendation was shortly (in November 2021) followed by the appearance of the novel SARS-CoV-2 variant—lineage B.1.1.529 (Omicron). While other variants termed Variants of Concern (VOC), had been characterized before [16], Omicron ignited special interest due to the significant reduction in vaccine effectiveness, as voiced in the Threat Assessment Brief by the European Centre for Disease Prevention and Control [17].

As it might have been expected, the visible, Omicron-related, increase in the number of cases stimulated interest in the third vaccine dose administration. The booster shot was offered in Poland to the entire adult population at the beginning of November 2021.

This study depicts the dynamics of the antibody response to the COVID-19 vaccination at multiple time points over a year from the first vaccine dose administration. The focus is set on the change in both anti-spike SARS-CoV-2 IgG and anti-nucleocapsid levels between 8 and 12 months after the first dose administration, affected by the virus variants spreading and booster dose administration. This late phase vaccine immunogenicity study may suggest some directions for the future vaccination strategies, including the crucial issue of further booster doses administration, especially in vaccinees with low concentration of anti-spike SARS-CoV-2 antibodies.

## 2. Materials and Methods

### 2.1. Study Design

The study participants (n = 100) were recruited from healthcare workers vaccinated against COVID-19 in the period of 4 January 2021–26 March 2021. The subjects were followed for around one year (median: 361 days) after the first vaccine dose administration.

The blood was drawn on day 0, 10 days after the first shot of the vaccine (day 10), before the second dose administration (day 20), at the peak of the response to the full vaccination (day 30), and then at the consecutive time points: days 60, 90, 120, 240 and 360 after the first dose administration.

The mean age of the study group was 45 years old (23–74), 89 subjects were under 60 years old. There was no statistically significant (Student’s *t*-test) difference in age between the sex subgroups (86% females and 14% males). All subjects received Pfizer-BioNTech Comirnaty vaccine, with two doses administered 21 days apart. Three out of the initial 100 participants were lost between days 240 and 360 of the study. Eighty out of 97 remaining participants chose to receive a booster shot of the Comirnaty vaccine. The booster was administered between days 241 and 341 of the study (median: 292 days after the first vaccination).

All subjects provided Informed Consent for the participation in the study and filled out a questionnaire about their history of COVID-19. Ethic approval of the study was obtained from Bioethics Commitee of Andrzej Frycz Modrzewski Krakow University, Krakow, Poland.

### 2.2. Laboratory Testing

The obtained samples were tested with 2 immunoassays: the LIAISON^®^ SARS-CoV-2 TrimericS IgG (DiaSorin S.p.A, Saluggia, Italy) and the Abbott’s SARS-CoV-2 IgG (Abbott, Sligo, Ireland). The former assay measures the concentration of the anti-spike SARS-CoV-2 IgG antibodies that are induced by both SARS-CoV-2 infection and COVID-19 vaccination. The latter detects the presence of the anti-nucleocapsid SARS-CoV-2 IgG antibodies, which are only elicited in the course of COVID-19.

The LIAISON^®^ SARS-CoV-2 TrimericS IgG (DiaSorin S.p.A, Saluggia, Italy) immunoassay was performed on fresh blood sera on the day of blood collection and the remaining sera were aliquoted and frozen (−20 °C) prior to testing with the Abbott’s assay. Both tests were performed strictly according to the manufacturers’ instructions.

The LIAISON^®^ SARS-CoV-2 TrimericS IgG assay was run on the LIAISON^®^ XL analyzer. This immunoassay allows for a quantitative measurement of IgG class antibodies against a trimeric spike (S) protein. The quantification is based on the chemiluminescent signal proportional to the concentration of the antibodies in the sample and expressed in Arbitrary Units (AU/mL). Owing to the correlation of the results of LIAISON^®^ SARS-CoV-2 TrimericS IgG to the values and units of the first WHO International Standard (IS) for anti-SARS-CoV-2 immunoglobulin binding activity (NIBSC 20-136) [18], the AU/mL may be converted to the Binding Antibody Units (BAU/mL) through a multiplication by a factor of 2.6. The assay’s cut-off for a positive result is 33.8 BAU/mL and the quantification range is between 4.81 and 2080 BAU/mL. The samples for which the results exceeded the upper quantification limit (UQL) were diluted 1:20, according to the manufacturer’s instructions, and re-tested.

The Abbott’s SARS-CoV-2 IgG is a chemiluminescent microparticle immunoassay (CMIA) for qualitative detection of IgG against the SARS-CoV-2 nucleoprotein. Nucleocapsid-specific IgG antibodies were tested to check for the possible unknown SARS-CoV-2 infection prior to the study, as well as for the contact with the virus over the course of the study.

The testing was performed on Abbott Architect i2000sr analyzer. The results are expressed as indices, calculated as a ratio of sample and calibrator signals. The threshold of positivity is 1.4.

### 2.3. Statistical Analysis

Student’s *t*-test was used to verify the statistical significance of the difference in age between the sexes. Mann–Whitney U test was used to verify the statistical significance of the difference in antibody concentration in sex and age groups and also to compare boosted vs. non-boosted groups. Wilcoxon test was used to compare the antibody concentrations at different timepoints. Kruskal–Wallis test was used to compare antibody concentration change in 4 subgroups, discerned by the booster and infection status. Post-hoc test with Bonferroni correction was included. Chi square test with Yates correction was used to verify dependency between booster administration and infection. Correlation between test results was assessed with r Spearman’s coefficient of rank correlation. The significance level was set to 0.05. The statistical analyses were performed with STATISTICA software ver. 13 (TIBCO Software Inc., Palo Alto, CA, USA).

## 3. Results

**Figure 1:**
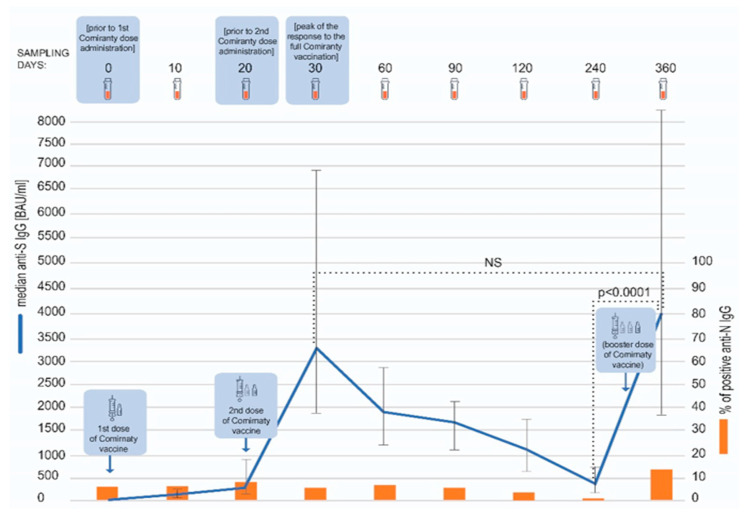
The model of the study. The concentrations of anti-spike SARS-CoV-2 IgG antibodies at different timepoints are presented as medians and interquartile ranges (Q25, Q75). Over the course of the study, anti-S concentrations peaked twice—after the second dose (day 30) and then after the booster (day 360) (blue line). The dotted lines indicate statistical significance of the differences in the antibody concentration between the investigated timepoints, as assessed with Wilcoxon test. The percentage of the subjects testing positive for anti-nucleocapsid SARS-CoV-2 IgG antibodies (orange bars) was relatively low throughout the study, but a rise was visible on day 360.

The dynamics of the humoral response to the COVID-19 vaccination was monitored for around one year (median: 361 days) after the first vaccine dose administration. We observed an expected pattern of the vaccination-elicited anti-spike SARS-CoV-2 response. The two-dose regimen resulted in a peaking concentration of specific IgG antibodies on day 30 (10 days after the second vaccine dose administration), with a median antibody concentration of 3500 BAU/mL (interquartile range 1880–6810 BAU/mL). Afterwards, a gradual decline was observed, with the lowest median concentration noted on day 240–379 BAU/mL (interquartile range 200–726 BAU/mL) (Figure 1). At this time point, only one vaccinee tested negative for anti-spike SARS-CoV-2 IgG.

Eighty participants received a booster shot of the Comirnaty vaccine. The third dose was administered between days 241 and 341 of the study (median: 292 day). On day 360, a sharp increase in the antibody titer, exceeding in magnitude the one noted on day 30, was observed. The median anti-spike SARS-CoV-2 antibody titer at this timepoint was 4070 BAU/mL (interquartile range 1870–8340 BAU/mL). The anti-spike SARS-CoV-2 IgG titers were statistically significantly higher on day 360 than 240 (*p* < 0.0001), but not between days 360 and 30 (*p* = 0.1676) (Figure 1), as assessed with Wilcoxon test. The antibody concentration on day 360 was not dependent on sex and age (below or over 60 years old) of the subjects (Mann–Whitney U test, *p* = 0.8792 and *p* = 0.7005, respectively). Additionally, we found no correlation between age and antibody concentration (Spearman’s rank correlation, R = 0.0632, *p* = 0.5379).

Over the course of the study, the participants were also tested for anti-nucleocapsid (anti-N) SARS-CoV-2 IgG (Figure 1). The presence of these antibodies indicates a natural contact with the virus. At the beginning of this study, 15 COVID-19 convalescents were included and 8 of them tested positive for anti-N IgG antibodies. Only one of these patients was still testing positive on day 360 whereas the rest became negative over the course of the study. In 100 fully vaccinated subjects, only 4 seroconversions were noted between days 60 and 120 (and none between 120 and 240) and all of these participants were again negative by day 240. In contrast, as many as 14 participants seroconverted in anti-N SARS-CoV-2 IgG between days 240 and 360 of the study.

Consequently, the substantial rise of the anti-S SARS-CoV-2 antibodies on day 360 might have been an outcome not only of the booster shot but also of the natural SARS-CoV-2 infection.

**Figure 2:**
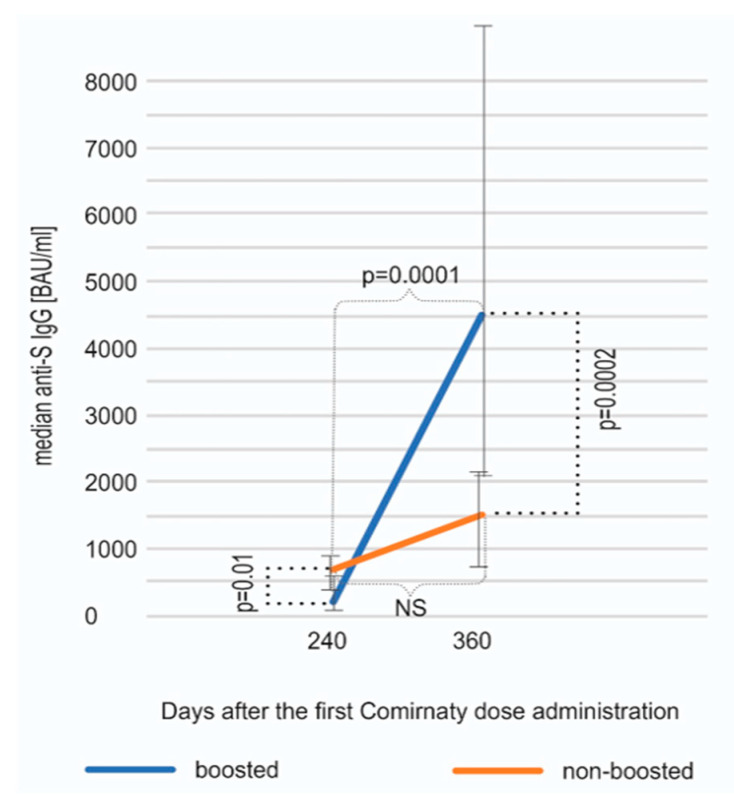
An increase in anti-spike SARS-CoV-2 IgG antibodies between days 240 and 360 shown separately for individuals boosted and non-boosted. The results are shown as medians and interquartile ranges (Q25, Q75). The rise was statistically significant only in the boosted subcohort (*p* = 0.0001, Wilcoxon test). On day 360, the boosted subjects displayed, as expected, a statistically significantly higher median anti-S IgG concentration (*p* = 0.0002, Mann–Whitney U test).

The median concentration of anti-S SARS-CoV-2 antibodies on day 360 was analyzed separately for the subgroups of individuals boosted (n = 80) and non-boosted (n = 17). Interestingly, in both of these subgroups, an increase between days 240 and 360 was observed; however, as it could have been expected, it was statistically significant only in the boosted subgroup (Wilcoxon test, *p* = 0.0001). The concentration of antibodies on day 360 was substantially higher in the boosted vs. non-boosted individuals (median 4670 vs. 1440 BAU/mL, *p* = 0.0002, Mann–Whitney U test) (Figure 2). The difference observed between these subgroups was statistically significant on day 240 as well (*p* = 0.01); however, interestingly, the level of antibodies on day 240 in the individuals who decided to reject the booster was higher than in vaccinees who chose to take the third dose. The subgroup of non-boosted participants included a higher percentage (35% vs. 15%) of individuals with COVID-19 history, which was probably the reason for the higher antibody titer and the decision on not taking the booster shot. As it could have been expected, the level of anti-S SARS-CoV-2 IgG on day 360 was much lower in the non-boosted group, obviously due to the lack of booster.

**Figure 3:**
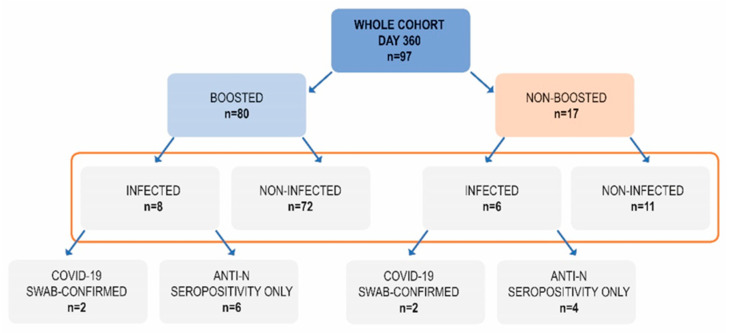
The composition of subgroups based on the booster administration and SARS-CoV-2 infection between days 240 and 360.

These two groups were divided further based on their recent (between days 240 and 360) SARS-CoV-2 infection, that was defined as an emerging anti-N seropositivity, in some cases confirmed by direct virus detection (Figure 3).

The largest group (n = 72) included participants who had received a booster but had not been infected between day 240 and 360 (boosted, non-infected; B, NI).

**Figure 4:**
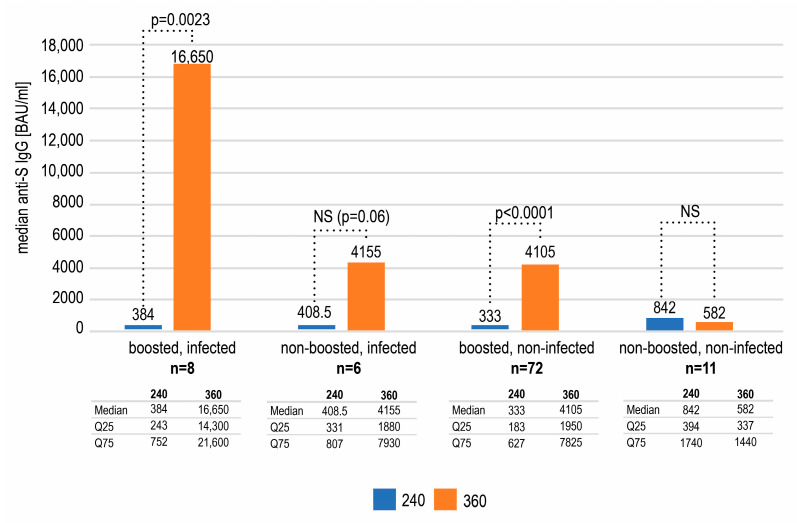
The median antibody concentrations and interquartile ranges on days 240 and 360 in subgroups defined by the recent infection and booster status. A statistically significant rise (Wilcoxon test) between those two timepoints was only seen in the subgroups of boosted subjects.

Out of 17 non-boosted subjects, as many as 35.3% had recently been infected. On the contrary, among 80 boosted subjects, there were only 10% who had recently been infected and this difference was statistically significant (*p* = 0.0206, Chi square test with Yates correction). Hence, this lower percentage may be attributed to the additional vaccine dose which was given at a median of 292 day—halfway between day 240 and 360. We could speculate that at least some out of eight infected subjects in the boosted group could have been infected before the booster. Retrospective analysis revealed that the non-boosted subjects who did not become infected between day 240 and 360 had had a higher median concentration of anti-S antibodies on day 240 (842 BAU/mL) than the non-boosted subjects who became infected—408 BAU/mL (Figure 4), although this difference was not statistically significant. Of interest, the subgroup of non-boosted, non-infected subjects included six who had had a history of COVID-19 (54% of the subgroup). Four of them had suffered from this disease before the first dose of vaccination and two had been infected after the primary inoculation regime but turned negative for anti-nucleocapsid IgG by day 120.

The comparison of the antibody concentrations on day 240 and 360 in the above mentioned four subgroups revealed an increase in the anti-S IgG in subjects that had been boosted and/or recently infected (Figure 4). The highest rise in the antibody concentration was seen in double-stimulated subjects. The increases observed in the subgroups of individuals only boosted or only infected were smaller but still substantial. The change between day 240 and 360 was statistically significant (Wilcoxon test) in subgroups of individuals boosted but not infected (*p* < 0.0001), as well as boosted and recently infected (*p* = 0.0023) but not in infected, non-boosted subjects (*p* = 0.06)—possibly due to the small number of subjects (n = 6). On the contrary, a further decrease, however insignificant (*p* = 0.1307), in the antibody titer was seen in the subgroup of participants that were neither boosted nor infected (–260 BAU/mL) (Figure 4).

**Figure 5:**
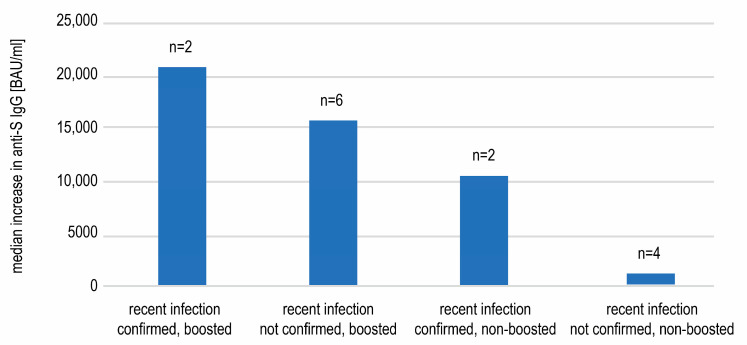
The median increases in the anti-spike SARS-CoV-2 IgG concentrations between days 240 and 360 observed in subgroups of recently infected individuals. The individuals with infection confirmed with a swab tended to display more pronounced rises in antibody titers.

Since anti-N seropositivity is only an auxiliary means of COVID-19 diagnosis, we researched if any of 14 recent convalescents in our study had had a proven record of COVID-19. We hypothesized that the concentrations of anti-spike antibodies may differ between convalescents confirmed with swab testing (which may indirectly indicate a more severe disease course) and those only anti-N seropositive. A positive result of either PCR or antigen testing of the nasopharyngeal swab sample was found for four subjects. We observed a higher increase in the concentration of anti-spike antibodies between days 240 and 360 in convalescents with confirmed SARS-CoV-2 infection in comparison to those only seropositive, in both boosted and not boosted individuals (Figure 5). The small number of subjects did not allow for these differences to be analyzed statistically.

**Figure 6:**
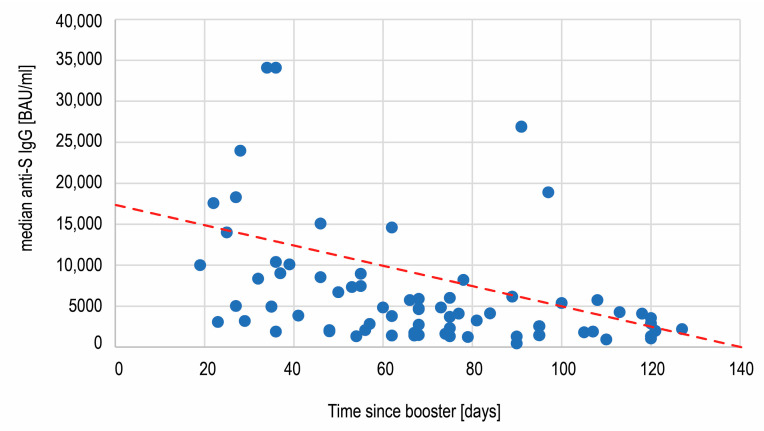
The negative correlation (R = −0.48, *p* < 0.00001) between the anti-spike SARS-CoV-2 IgG concentration and time passing after the third Comirnaty dose administration.

The wide range (434–34,100 BAU/mL) of the antibody titers on day 360 observed in the biggest subgroup of boosted but not infected individuals was examined further. A statistical analysis revealed that the antibody concentration was not dependent on age (below or over 60 years old) and sex of the subjects (Mann–Whitney U test, respectively, *p* = 0.9051 and *p* = 0.9830). Additionally, we found no correlation between age and antibody concentration (Spearman’s rank correlation, R = 0.0328, *p* = 0.7847). A correlation between the anti-S SARS-CoV-2 IgG concentration and the number of days between the booster shot and the measurement on day 360 was assessed (Figure 6). A statistically significant negative correlation (Spearman’s rank correlation) between these variables was revealed (R = −0.4789; *p* < 0.00001), showing the decrease of antibodies with time after vaccination.

## 4. Discussion

This study followed COVID-19 vaccinees over one year. The interim results, picturing the dynamics of the humoral response of 100 volunteers to the vaccination up to 8 months after the first dose administration had already been published [10,19]. In the first paper [19], we reported the substantial rises in the anti-spike (S) SARS-CoV-2 IgG antibody concentrations following both Comirnaty doses administrations, with the peak response being noted 10 days after the second vaccine shot. Afterwards, anti-S IgG gradually declined [10]. A similar pattern in Comirnaty vaccine recipients has been observed, e.g., by Salvagno et al. [20]. It must be noted, that in our cohort the median antibody titer (379 BAU/mL) on day 240 (8 months after initial immunization) was still more than 10 times higher than the assay’s positivity cut-off and only one participant tested negative for anti-spike SARS-CoV-2 IgG. In addition, it was noted that 65% of the subjects in our study had a concentration higher than 264 BAU/mL, indicating 80% COVID-19 vaccine efficacy against symptomatic disease, as determined by Feng et al. [21]. Our observations are in line with the communications from other research groups that described the decrease in the humoral immunity that starts as early as one month after the vaccination and continues over the next 9 months [22,23].

The current study extends the previous observations on the antibody titers over the period between 8 and 12 months after the vaccination. We demonstrate that the anti-spike IgG concentration is determined by the status of the booster shot administration and the history of recent COVID-19.

The booster dose administration was recommended by the WHO and subsequently other authorities such as the European Commission in response to the reports on the waning humoral immunity, followed by the emergence of the new SARS-CoV-2 variants. In Poland, this opportunity arose between days 240 and 360 of our study and was manifested by a substantial increase in the median concentration of the anti-S SARS-CoV-2 IgG antibodies between those two time points (*p* < 0.0001). The observed peak was higher than the one noted after the primary immunization, which is what has already been reported by other authors [20,23,24]; however, this difference was not statistically significant (*p* = 0.17). Of note, as much as 82% of the participants decided to receive a booster shot of the Comirnaty vaccine. A similar percentage (70%) has been reported by Chivu-Economescu et al. [25], which proves a high level of confidence in the immunity conferred by the COVID-19 vaccination.

The results of our study indicate that a booster dose is the most powerful single measure accessible to increase anti-spike IgG concentration. This is important, since this type of antibodies has been reported to confer the neutralizing function and hence has been considered one of the correlates of immunity against SARS-CoV-2 infection [6,21]. There are a number of reports showing the relationship between the antibody presence and titer and their neutralizing potential and ultimately COVID-19 protection [21,26,27,28]. The general agreement is that the higher antibody titer indicates a better protection, at least against severe infection [21].

A statistically significant rise in anti-spike IgG concentration between day 240 and 360 was noted in our study only in the subgroups of boosted individuals. However, in the non-boosted but recently infected subgroup, a statistically insignificant increase was also seen.

The stimulating effect of the recent natural infection in our study was evident in the small subgroup of boosted and also recently SARS-CoV-2-infected participants, who displayed the highest increase between days 240 and 360 (median increase 16266 BAU/mL). This finding may suggest that these two types of immunization work synergistically. Lee et al. have also reported a higher level of anti-S antibodies 4 months after booster in subjects infected with SARS-CoV-2 within this period in comparison to the boosted participants without SARS-CoV-2 infection. The phenomenon named hybrid immunity—stemming from combined vaccination and infection, has been linked to a higher protection against SARS-CoV-2 and its variants, and explained by the induction of more broadly neutralizing antibodies and a stronger cellular immune response [25,29,30]. Lee at al. have also reported that the level of anti-nucleocapsid SARS-CoV-2 antibodies, indicating contact with the virus, correlated positively with the concentration of anti-spike IgG [31]. Similar analysis performed in our study did not reveal a statistically significant correlation between anti-N levels and anti-S concentration (data not shown).

As could have been expected, in the non-stimulated by either vaccination or recent infection subjects, a further (though statistically insignificant) median decrease in the antibody titer between days 240 and 360 was observed. Of interest, this subgroup included six subjects with COVID-19 history (54% of the subgroup), and their convalescence could have been the reason for the highest median anti-spike IgG concentration in this subgroup on day 240. Importantly, those subjects did not become infected over the following four months (between days 240 and 360), and it may be speculated that the high antibody concentration contributed to their COVID-19 immunity. However, past COVID-19 infection was not enough to sustain the antibody titers, which decreased in this subgroup on day 360.

Data from our study brings some additional interesting observations. Twelve of the seventeen individuals who opted out of the booster vaccination had had a history of COVID-19, which might have been the justification for their decision. However, 35% of the non-boosted subjects became infected with SARS-CoV-2 between days 240 and 360, in comparison to only 10% in the boosted group. The comparison of the antibody titers revealed insignificantly lower anti-S IgG concentrations prior to infection in the non-boosted, subsequently infected subjects. Though no unequivocal protective titer has been established, the manufacturer-provided data correlates anti-trimeric spike concentration of 520 BAU/mL with the microneutralization titer of 1:80, and the abovementioned paper by Feng et al. tentatively points to the concentration of 264 BAU/mL as conferring protection. Of note, the median antibody concentration on day 240 in our subgroup of non-boosted, non-infected individuals (842 BAU/mL) was higher than both these numbers. This may indicate that the anti-spike antibody concentration should be taken into account while making the decision on booster dose administration.

The main aim of COVID-19 vaccination is to prevent severe disease and to lower hospitalization rates. Anti-nucleocapsid IgG are detected after infection but not after mRNA-based vaccination and correlate with the severity of the disease, with the higher levels and longer persistence being observed after a more serious COVID-19 course [32,33]. The infections reported in vaccinated subjects during our study seem to be rather mild—the anti-N antibodies are detected only for up to 3 months in individuals infected in the middle of the study course (between second and third dose), and the appearance of anti-N IgG on day 360 in 10 out of 14 subjects was discovered solely because of the serological testing confined to the participation in the study, and not by symptoms or direct swab testing. Four subjects with COVID-19 confirmed with swab testing, which might imply a more serious disease, showed a higher increase in anti-spike antibody titers between days 240 and 360.

Therefore, our results add to the body of data supporting the COVID-19 vaccination. However, the protection conferred by the vaccination decreases over time. During 8 months after the primary immunization, only 4 out of 100 fully vaccinated subjects seroconverted. On the contrary, over the following 4 months, 14 participants developed anti-N antibodies. The observed increase is in line with Mizrahi’s observation on the correlation between time-from-vaccine and incidence of breakthrough infections [34] and clearly mirrors the fading anti-spike SARS-CoV-2 antibody concentrations. The appearance and spreading of the Omicron, a new variant of SARS-CoV-2, could have been an additional reason for the higher rate of the infections, independently from the waning of the immunity [35].

Between days 240 and 360 of our study, the majority (n = 72) of the subjects had received the third dose of the Comirnaty vaccine and had not been infected with SARS-CoV-2. The anti-spike concentrations measured in individuals in this subcohort on day 360 differed substantially (between 434 and 34,100 BAU/mL) and did not depend on age and sex of the subjects. We found that the time passing after the booster influenced the antibody concentration—there was weak, but statistically significant negative correlation between these two variables. Some reports have already been published on the waning vaccine effectiveness against COVID-19 with time since boosting [36] and a quick antibody level decrease after the booster vaccination [23,25,29]. For example, in a study by Ogric et al., anti-S1 SARS-CoV-2 IgG concentration decreased 2.5x between measurements 3 weeks and 3 months after the booster in naïve vaccinees. In our study, on day 360 the non-infected individuals who had received the third Comirnaty dose still demonstrated anti-S antibody median concentration 12.3 higher than on day 240 (4105 vs. 333 BAU/mL, respectively). This proves that at least in some vaccinees the effect of a booster shot is sustained over a few months (the subjects were tested at a median of 68 days after the booster) and, therefore, may be expected to reduce the rates of both confirmed and severe COVID-19 [37,38], especially in light of the findings that it enforces protection against SARS-CoV-2 variants [39,40].

Data published thus far on the influence of age and sex on the humoral response to COVID-19 vaccination indicates that females and younger subjects display higher levels of anti-spike SARS-CoV-2 antibodies [41,42,43,44]. Previously we have reported that females tended to develop higher antibody titers than males, but the differences were only statistically significant between days 60 and 120 after the first vaccination [19]. However, on day 360 males exhibited statistically insignificantly higher median titers (4105 BAU/mL vs. 3840 BAU/mL in females). When the influence of sex was analyzed separately for boosted, not infected individuals, the difference between males and females on day 360 was negligible (respectively median 4105 and 4170 BAU/mL). In addition, in our cohort younger subjects (below 60 years old) displayed statistically significantly higher antibody concentrations only at the primary peak of the immune response after the second vaccine dose administration (day 30), which could have been caused by the less dynamic antibody production in the older subjects, reported by other authors as well [45]. Later, the anti-S SARS-CoV-2 IgG concentration did not differ significantly between the age groups. This was also observed on day 360, both in the whole cohort and in the subgroup of boosted, non-infected individuals. Although, it must be noted that females and participants below 60 years old were overrepresented in our cohort, which might have influenced the significance of the results presented here.

Another limitation of some of the observations reported in this paper are the small sizes of the subgroups, especially of non-boosted and recently infected individuals. Thus, the interesting finding in the non-boosted group—the higher antibody concentration in subjects not infected over the following 4 months in comparison to the infected ones—should be interpreted with caution and requires further confirmatory studies.

## 5. Conclusions

The results of our study indicate that the booster immunization is the most efficient way of stimulating production of anti-spike, potentially neutralizing antibodies. The level of anti-spike antibodies was statistically higher in the boosted subgroup and in this subgroup a lower percentage of infections was noted. The humoral immunity is additionally enhanced by the natural contact with SARS-CoV-2, as the highest antibody concentration was seen in the boosted and infected subgroup. However, infection without a booster resulted in only insignificant increase in anti-spike concentration. We provide some hints on the usefulness of anti-spike antibody testing prior to additional vaccine dose administration, as higher antibody concentration was seen in subjects not infected over the next four months. Thus, in individuals with low level of antibodies, booster dose administration may be crucial. Further research is needed to elucidate whether antibody titers are truly indicative of COVID-19 immunity and to provide method-dependent protective anti-spike SARS-CoV-2 IgG concentrations.

## Data Availability

The data presented in this study are available on reasonable request from the corresponding author.
